# Biologic Monitoring of Exposure to Environmental Chemicals throughout the Life Stages: Requirements and Issues for Consideration for the National Children’s Study

**DOI:** 10.1289/ehp.7617

**Published:** 2005-05-12

**Authors:** Dana B. Barr, Richard Y. Wang, Larry L. Needham

**Affiliations:** National Center for Environmental Health, Centers for Disease Control and Prevention, Atlanta, Georgia, USA

**Keywords:** adducts, adipose tissue, bioaccumulative, biomonitoring, blood, breast milk, matrix, metabolite, National Children’s Study, nonpersistent, persistent, toxicant, urine

## Abstract

Biomonitoring of exposure is a useful tool for assessing environmental exposures. The matrices available for analyses include blood, urine, breast milk, adipose tissue, and saliva, among others. The sampling can be staged to represent the particular time period of concern: preconceptionally from both parents, from a pregnant woman during each of the three trimesters, during and immediately after childbirth, from the mother postnatally, and from the child as it develops to 21 years of age. The appropriate sample for biomonitoring will depend upon matrix availability, the time period of concern for a particular exposure or health effect, and the different classes of environmental chemicals to be monitored. This article describes the matrices available for biomonitoring during the life stages being evaluated in the National Children’s Study; the best biologic matrices for exposure assessment for each individual chemical class, including consideration of alternative matrices; the analytical methods used for analysis, including quality control procedures and less costly alternatives; the costs of analysis; optimal storage conditions; and chemical and matrix stability during long-term storage.

After an individual’s exposure to a given chemical, a proportion of the chemical may be absorbed into the bloodstream, distributed among the bodily tissues, metabolized, and/or excreted. These four complex steps [i.e., absorption, distribution, metabolism, and excretion (ADME)] make up the pharmaco-kinetic process of a chemical (reviewed in Rozman and Klaasen 2001). In order to assess human exposure to a given chemical, biologic measurements of the chemical can be made after the absorption step or during each of the subsequent steps of ADME. Biomonitoring of exposure involves the measurement of the concentration of a chemical in a given biologic matrix during or after ADME, and its concentration level depends on the amount of the chemical that has been absorbed into the body, the pharmacokinetics (ADME) of the chemical, and the exposure scenario, including the time sequence of exposure and time since last exposure (Sexton et al. 1995). Biomonitoring data are independent of the pathway of exposure (Pirkle et al. 1995). Ideally, in order to link the dose with adverse health outcomes, measurements of the biologically effective dose, the dose at the target site that causes an adverse health effect, are preferred (Pirkle et al. 1995). However, often the target organ is not known and, even if known, frequently is not available for sampling. In these situations, we measure the level of the chemical in another biologic sample to gauge the internal dose.

For the National Children’s Study (NCS 2005), the biologic sample can be taken preconceptionally from both parents; from a pregnant woman during each of the three trimesters, during and immediately after childbirth; from the mother postnatally; and from the child as it develops up to 21 years of age. The appropriate sample for monitoring will depend upon matrix availability and the different classes of environmental chemicals to be monitored. Under the auspices of the Chemical Exposures Workgroup of the NCS, we developed this article as part of a larger white paper (NCS 2004) to provide guidance on which biologic samples may be most useful for characterizing exposures of interest in the NCS. Although this guidance may be applicable to other exposure studies, it was developed with the life stages of interest to the NCS and with the recognition that the specimens available for testing may be limited in volume or quantity (Needham et al. 2004, 2005). Unless otherwise stated, we refer to measurements made on biologic samples from the parents or the child but not from the fetus. Further, we focus primarily on chemical measurements made in a biologic matrix that is taken from the participant, a commonly used strategy in human exposure assessment. Although newer methodologies such as imaging techniques and “omics” technology are becoming more readily available (Chaussabel 2004; de Hoog and Mann 2004; Dettmer and Hammock 2004; Kiechle et al. 2004; Olden 2004; Wetmore and Merrick 2004), they are not included in this article.

## The General Behavior of a Chemical in the Body

Absorption of a chemical into the body occurs when the chemical enters the bloodstream by passing through absorption membrane barriers after contact of the chemical with an outer boundary (i.e., skin, nostrils, mouth, or eyes). Without absorption, there can be no direct internal toxic effect even if the chemical is toxic, although effects are possible at the absorption barrier (e.g., skin irritation, eye lens irritation). Once the chemical has been absorbed into the bloodstream, it undergoes distribution to the primary deposition sites. Distribution is crucial to toxicity because if the chemical is never distributed to the target site, the toxic effect may be negligible. The concentration of the chemical in the storage depot is in equilibrium with the concentration in the blood, thus the chemical is slowly released from the storage depot as it is eliminated from the blood to maintain the equilibrium. Low concentrations may reach the target organ.

Metabolism takes place primarily in the liver. The overall purpose of metabolism is to make the chemical less toxic and more hydrophilic. Phase 1 metabolism of the chemical typically involves inserting or substituting a functional group to make the chemical more water soluble. Phase 2 metabolism usually chemically links the chemical to a glucuronide or sulfate group, which increases the water solubility and facilitates elimination of the chemical in the urine. However, metabolism does not always render a chemical less toxic.

Metabolized chemicals may be more hydrophilic and can be excreted in urine or may be passed into the feces. If the chemical is not absorbed, it can go straight into the feces. Lipophilic compounds, in particular, are eliminated primarily in the feces. Volatile organic compounds (VOCs) can be excreted through the alveoli or in the expired air through exhalation. Chemicals can also be deposited in certain secretory structures and be excreted as tears, saliva, sweat, or milk in lactating women.

In addition to the internal movement of chemicals in the body, a pregnant woman can distribute the chemicals via the bloodstream through the placenta and into the fetal blood supply. Biomonitoring matrices unique to the fetus include amniotic fluid and meconium. In addition, cord blood, the placenta, and the umbilical cord can be collected at birth.

## Behavior of Specific Chemical Classes in the Body

### Persistent organic chemicals.

Persistent organic pollutants or chemicals (POPs) include polychlorinated dibenzo-*p*-dioxins, polychlorinated biphenyls (PCBs), and organochlorine insecticides (Needham et al. 2005; United Nations Environment Program 2001). Polycyclic aromatic hydrocarbons (PAHs) are also often included in this class because they persist in the environment; however, because PAHs behave more like nonpersistent chemicals in the body, we have chosen to exclude them from POPs (Needham et al. 2005). The primary route of exposure to POPs is ingestion. POPs are readily absorbed into the blood supply by passive diffusion. Their blood level initially decays relatively rapidly, representing the alpha decay period (Flesch-Janys et al. 1996). During the alpha decay, the POP is distributed into the fatty portions of tissues and, in lactating women, in breast milk. The concentration of the POP in the fatty portions of tissues is in equilibrium with the concentration in the lipid portion of blood. The fat content of blood serum is 0.5–0.6%, milk is approximately 4% lipid, and adipose tissue may be as much as 95% lipid. Thus, although the equilibrium concentrations of the chemical in the blood and fatty tissues may differ over orders of magnitude, they may be very similar when matrices are adjusted for lipid content.

In pregnant women, the POP may also distribute in the fetal compartment; therefore, other matrices such as cord blood or serum may be used for POP measurements. However, the lipid content of cord blood is lower than that of an adult’s blood, so the sensitivity of the analytical measurement may play a key role in obtaining a valid measurement in cord blood. Other fetal matrices, such as meconium, have not been fully explored for their potential in assessing POP exposures in the fetus. Maternal blood or adipose tissue taken before or during pregnancy and maternal blood, milk, or adipose tissue taken soon after parturition (if breast-feeding or can be taken later if not breast-feeding) are considered the best matrices for estimating fetal exposures to POPs.

Because metabolism and excretion of POPs are very slow, they have a long half-life in the body, usually along the order of years (Michalek et al. 1996; Phillips et al. 1989b). However, because the lipophilic POPs accumulate in the breast milk of lactating women and the milk is removed from the woman’s body, the half-life of POPs in lactating women is about 6 months (LaKind et al. 2000).

### Nonpersistent organic chemicals.

**Figure 1:**
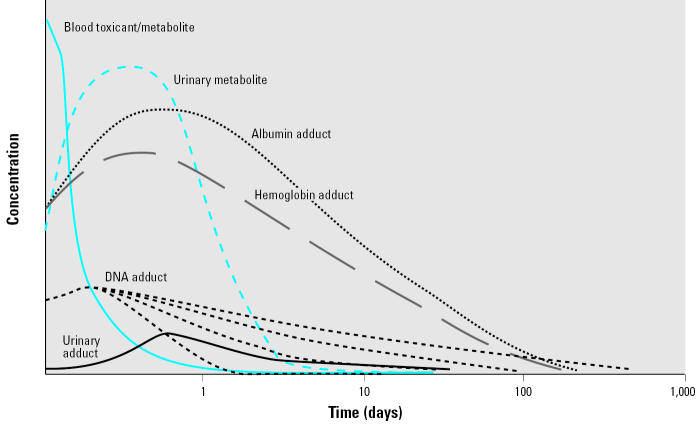
Hypothetical postexposure fate of a nonpersistent toxicant in blood and urine. Reproduced from Needham and Sexton (2000) with permission of Nature Publishing.

Nonpersistent organic chemicals, such as current-use pesticides, phthalates, and VOCs (Needham et al. 2005), can be much more challenging to measure. Their primary routes of exposure for the general population, depending on the scenario, are generally ingestion or inhalation. These chemicals are rapidly metabolized, and their metabolites are eliminated in urine (Figure 1). The deposition matrices are minor matrices for monitoring because only small amounts of the chemical are deposited in the body. The major matrices for assessing exposure are excreta. Blood has also been used as a matrix for biomonitoring. Nonpersistent chemicals tend to have very short half-lives in blood (Figure 1), and the concentrations are usually about three orders of magnitude lower than urinary metabolite levels (Barr et al. 1999). Thus, if blood is used as a matrix, the sensitivity of the analytical method and the matrix volume available for analysis may become important. Blood can also be a valuable matrix for measuring biomolecular adducts such as hemoglobin, albumin, or DNA adducts, such as DNA–PAH adducts.

Saliva has also been explored as a matrix for measuring selected nonpersistent chemicals such as atrazine (Lu et al. 1998). The existing data indicate that saliva levels can be considerably lower than blood levels of a nonpersistent chemical depending upon the degree of protein binding that may occur; thus, a very sensitive analytical technique is required. Further research on additional chemicals and the relation of these measurements to more commonly used approaches is required before this can routinely be used for analysis.

**Figure 2:**
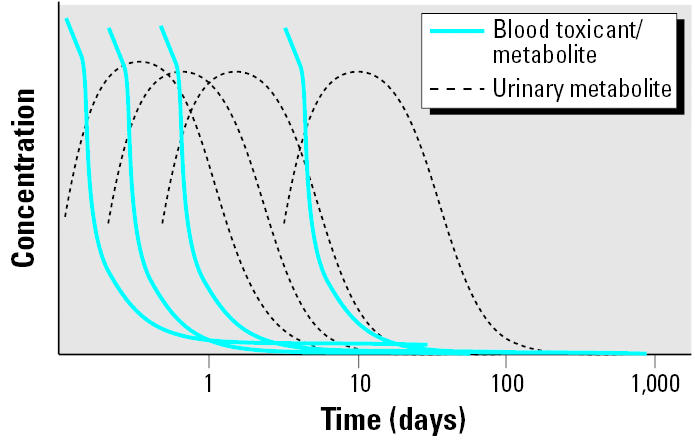
Hypothetical postexposure fate from chronic exposure to a nonpersistent toxicant in blood and urine.

To evaluate fetal exposures, maternal samples collected throughout pregnancy may be used. However, because these chemicals are, by definition, nonpersistent, urine or blood measurements made at a single point in time during pregnancy will address only the exposures that may have occurred in the previous few days unless the exposure is continuous (e.g., pervasive air levels of a chemical resulting from smokers in the home) or continual (e.g., eating the same foods daily with measurable levels of pesticides) (Figure 2). To circumvent this problem, multiple biologic samples can be taken every few days during pregnancy; however, this can be costly and logistically difficult to collect and store and may present an undue burden on the participant. An alternative may be to collect multiple samples over particularly vulnerable stages of the pregnancy, if such stages can be appropriately identified. Another potential approach is to measure nonpersistent chemicals in fetal matrices such as cord blood or meconium.

### Bioaccumulative metals.

Bioaccumulative metals persist in the environment and bio-accumulate in humans. This group of chemicals includes some forms of mercury, lead, and cadmium (Needham et al. 2005). For example, lead is readily absorbed, particularly in children, with distribution from the blood to its storage depots—bone and teeth (Aufderheide and Wittmers 1992). Both metabolism and excretion are slow, so monitoring lead levels is more straightforward. The best matrices to use are blood, bone, and teeth. For general population exposures to mercury, methyl mercury is the form of highest concern. Blood, hair, and nails are viable matrices for measuring methyl mercury levels.

### Nonbioaccumulative metals.

Non-bioaccumulative metals are readily absorbed into the body, and although some proportion may distribute to various tissues, most will pass through the body rapidly. These metals are typically measured in urine (Horng et al. 1999). However, to gain a longer term dosimeter for exposure, arsenic can also be measured in hair (Wilhelm and Idel 1996) and nails (Lin et al. 1998).

### Criteria pollutants and bioallergens.

In general, biomonitoring has a limited role in the measurement of criteria pollutants [e.g., carbon monoxide (CO), oxides of nitrogen, ozone] and bioallergens (e.g., pollen, endotoxins) (Needham et al. 2005). Exposure to CO can be assessed by measuring the carboxyhemoglobin adduct (Shenoi et al. 1998; Smith et al. 1998) or expired CO (Lapostolle et al. 2001; Paredi et al. 2002) in blood and breath, respectively. The adduct measurements provide a longer-term dosimeter for the exposure than breath measurements because hemoglobin has a lifetime of about 4 months.

Bioallergen response can be measured by IgE in maternal, cord blood, or child blood (Carrer and Moscato 2004; Goodman and Leach 2004; Lee et al. 2004). In addition, certain endotoxins or metabolites may be measured in blood or urine samples (Makarananda et al. 1998; Malir et al. 2004). Typically, the endotoxin measurements would reflect a more recent exposure, similar to nonpersistent chemical exposures.

## Assessing Exposure throughout the Life Cycle

Biomonitoring measurements have been used for many years to assess exposures in adults (Ashley et al. 1994; Blount et al. 2000; CDC 2003; Pirkle et al. 1995) and, to some extent, in adolescents and children (Adgate et al. 2001; Fenske et al. 2000). Biomonitoring of fetuses, infants, and small children has been performed much less frequently, if at all. Various biologic matrices have been used or considered for assessing environmental exposures throughout the life cycle (Table 1). The mother or pregnant woman has generally been used as a surrogate to evaluate fetal exposures. However, for many chemicals, their ability to transfer from the mother to the fetus is not known and the relationship between maternal and fetal chemical levels has not been defined. Another potential option to evaluate fetal exposures is the use of meconium as a matrix of measurement because it begins accumulating in the bowels of the infant during the second trimester (Bearer 2003; Bearer et al. 2003; Ostrea et al. 1994). However, meconium use has many limitations. Meconium measurements are still in their infancy of development, and to date, no reliable way to relate these measurements to measurements in more commonly used matrices (e.g., urine, blood) exists. In addition no information is gleaned from exposures that occurred in the first trimester. However, many meconium measures have been shown to correlate well with reported maternal exposures to tobacco (Ostrea et al. 1994), drugs of abuse (Ostrea 1999), and alcohol consumption (Bearer et al. 2003), and this matrix shows promise for other chemical exposures of concern (Whyatt and Barr 2001).

The period from birth through 1 year of age is also very important (Needham and Sexton 2000; Needham et al. 2004, 2005). During this time the infants may be breast-feeding, so they may be exposed to chemicals via breast milk. In addition their micro-environments are often close to the floor and substantially different from an older child or adults. At this age probably only urinary chemical measurements and breast milk measurements can be made. Urine volume will likely be limited, usually 10 mL or less.

Once children start school, another environment with potential chemical contamination is included in the exposure scenario; however, biologic sample collections become easier. At this stage in life some blood can be collected, but it is often limited to a small amount. Urine and saliva samples also can be readily collected. As children approach adolescence and adulthood, more biologic samples and/or a greater quantity of a matrix can be collected. At this life stage perhaps up to 100 mL of blood can be collected for various measurements, and urine is typically plentiful.

## Biologic Matrices for Exposure Assessment

The two primary matrices used to assess human exposure to chemicals are urine and blood (serum, plasma, blood cells, etc.) (Barr et al. 1999; Needham and Sexton 2000; Pirkle et al. 1995).

### Blood.

Many persistent and nonpersistent chemicals can be measured in blood (Angerer 1988; Angerer and Horsch 1992; Barr et al. 2002; Leng et al. 1997). Although the amount of blood is similar in all adults, the chemical composition of blood, such as lipid content, varies between individuals and within an individual, especially postprandial (Phillips et al. 1989a). Blood concentrations of lipophilic chemicals are routinely normalized using blood lipid concentrations to allow a direct comparison of their concentrations within and among individuals, regardless of the time of day the blood was collected. However, other chemicals that can be measured in blood may not vary based upon the blood lipid content. For example, fluorinated chemicals in blood are not dependent upon the lipid content; instead, they bind to blood albumin (Jones et al. 2003). Therefore, these measurements should not be adjusted based upon the blood lipid content; however, other adjustments, such as for albumin content, may be required if deemed appropriate.

Measuring a chemical in blood is inherently advantageous (Barr et al. 1999). Because we know how much blood is in the body, we can calculate the body burden more accurately than if we measure the chemical or its metabolite in urine. However, blood collection is invasive, which may severely limit the ability to collect it from infants and small children. In addition nonpersistent chemicals are usually found in very low concentrations in blood (Barr et al. 1999, 2002). Also, if testing is not performed soon after sample collection, which will likely be the case in the NCS, long-term storage of blood may be problematic, depending upon what form of blood is being stored. Storage conditions and stability of various matrices and chemicals are shown in Table 2.

### Urine.

One major advantage of using urine in biomonitoring is the ease of its collection for spot urine samples (Barr et al. 1999; Needham et al. 2004); however, the collection of 24-hr urine voids can be very cumbersome and result in nonadherence (Kissel et al. 2005). Therefore, spot urine samples, whether first-morning voids or “convenience” samplings, are most generally used for biomonitoring purposes. The major disadvantages of spot urine samples include the variability of the volume of urine and the concentrations of endogenous and exogenous chemicals from void to void (Barr et al. 1999; Kissel et al. 2005). The issue on how best to adjust the urinary concentrations of environmental chemicals in a manner analogous to the adjustment of the concentrations of lipophilic chemicals in blood is a subject of continued research. Adjustment using urinary creatinine concentrations [i.e., dividing the analyte concentration by the creatinine concentration (in grams creatinine per liter urine)] is the most routinely used method for correcting for dilution. Analyte results are then reported as weight of analyte per gram of creatinine (e.g., micrograms analyte per gram creatinine). This may work well when comparing analyte levels in a single individual because the intraindividual variation in creatinine excretion is relatively low; however, for diverse populations the interindividual variation is extremely high (Barr et al. 2005).

### Breast milk and adipose tissue.

Many of the chemicals measured in blood have been found in breast milk (LaKind et al. 2001) and adipose tissue (Patterson et al. 1987). Breast milk measurements are unique in that they not only provide data on ingestion exposures for the infant but also are indicators of maternal exposures. Breast milk and adipose tissue are lipid-rich matrices, more so than blood, so similar lipid adjustments are required for reporting concentrations of lipophilic analytes. In general these lipophilic analytes partition among the lipid stores in blood, breast milk, and adipose tissue on nearly a 1:1:1 basis (Patterson et al. 1987). More laboratory work needs to be done on the partitioning of less bioaccumulative analytes in these matrices.

### Alternative matrices.

Chemicals have been successfully measured in alternative matrices such as saliva (Bernert et al. 2000; Lu et al. 1998), meconium (Bearer et al. 1999, 2003; Ostrea 1999; Ostrea et al. 1994; Whyatt and Barr 2001), amniotic fluid (Bradman et al. 2003; Foster et al. 2002), and breath (Pellizzari et al. 1992). Because many of these matrices are not commonly analyzed, the resulting chemical concentration data are more difficult to relate to measurements made in the more commonly used matrices such as urine, blood, or breast milk and, consequently, may be more difficult to relate to exposure. However, because many of these matrices are available and could provide potentially useful information, they should not be discounted. Instead, preliminary studies evaluating the partitioning of chemicals in the various matrices should be conducted that will allow for comparison of data among matrices.

### Measurement method specificity and sensitivity requirements.

Specificity—how specific an analysis method is for a particular exposure—and sensitivity—the ability to measure the chemical at the desired level—are critical parameters for analysis methods, and both must be considered when deciding which matrix to measure. The half-life of a chemical may affect the sensitivity requirement; however, because persistent chemicals have long half-lives, it is not nearly as important as it is for nonpersistent chemicals, which metabolize rapidly. For instance, in adult men, 2,3,7,8-tetrachlorodibenzo-*p*-dioxin has a half-life of about 7.6 years (Pirkle et al. 1989). Therefore, to assess exposure over a period of time, for example, 9 months, the sample could be collected at any time period within the 9 months or even afterward, and the biologic measurement information would still be useful for accurate exposure classification (e.g., exposure quartiles—people whose exposure is high, medium, low, or none). When measuring exposure to persistent chemicals by analyzing adipose tissue, it makes little difference which portion of the body the sample is taken from; however, because blood is easy to collect and readily available, blood is an ideal medium in which to measure persistent chemicals. In lactating women, milk is also frequently used.

Nonpersistent chemicals have half-lives of hours or minutes; therefore, the postexposure fate of a nonpersistent chemical is dramatically different (Figure 1) (Needham and Sexton 2000). After each exposure the concentration of the chemical in blood declines rapidly. The window of opportunity for measuring nonpersistent chemicals in blood is narrow and requires the use of a very sensitive technique. By measuring these chemicals in blood as the intact, or parent, chemical, we gain information on the exact chemical to which one was exposed. For example, if someone was exposed to chlorpyrifos, we can measure chlorpyrifos in the blood rather than its metabolite, which is formed from more than one parent chemical and is also the same chemical as environmentally degraded chlorpyrifos. In addition to blood, certain nonpersistent chemicals such as cotinine have been measured in saliva because cotinine is in equilibrium in blood and saliva.

In urine we generally measure metabolites of the chemical that may lack the desired specificity for analysis; however, measurements in urine allow a much wider window of opportunity in which to take the sample. Generally, we assess exposure to nonpersistent chemicals by measuring their metabolites in urine, even though this method may not have the specificity of the blood measurement.

When chronic exposure to a nonpersistent chemical occurs, the exposure is continually replenishing the chemical in the blood and urinary elimination may reach a steady state. Therefore, urine becomes a better matrix for measurement because we integrate exposure over a longer period.

### Biomolecular adducts.

Persistent and nonpersistent chemicals can also react with bio-molecules such as DNA, hemoglobin, or fatty acids to form biomolecular adducts (Angerer et al. 1998; Schettgen et al. 2002). By measuring these adducts, we are able to increase the amount of time after exposure that we can measure a nonpersistent chemical because the amount of time the adduct remains in the body is largely dependent upon the lifetime of the biomolecule itself (Needham and Sexton 2000). For example, the average life span of a red blood cell is about 120 days. If a chemical formed an adduct with hemoglobin on the day a red blood cell was created, the adduct should remain in the body about 4 months, allowing a much longer time after exposure to collect the sample. Other adducts are formed with DNA, albumin, and other prominent proteins. Because adducts are not formed from every chemical molecule to which one is exposed, adduct measurements must be very sensitive, and usually a large amount of matrix is required. In addition the measurements are usually cumbersome and time-consuming, so the analytical throughput is very low and the cost is very high.

When measuring persistent chemicals, we do not gain much advantage by measuring them as adducts. Blood is still the matrix of choice because the concentration is higher in blood, and we have a wide window of opportunity (Barr and Needham 2002). To form an adduct, the chemical must have an electrophilic site to which a nucleophile on the biomolecule (usually sulfur or nitrogen) can covalently bind.

### Sampling time frame.

For persistent organic chemicals, the time frame for sampling is reasonably straightforward. In general, a blood sample can be taken any time, up to several years after exposure, has occurred and the exposure can still be accurately identified; however, the investigator will not have any information about when the exposure occurred. For example, if a PCB concentration of 1,000 ng/g lipid was measured in a blood sample, it is not known if a recent exposure to this amount of PCB occurred or whether a larger exposure occurred many years ago and, although a portion of the PCB has been eliminated from the body over time, this amount is still circulating in the bloodstream. By coupling questionnaire data with these biologic measurements, investigators may gain information on the timing of the exposure (e.g., breast-feeding, subsistence food consumption).

The sampling time frame for nonpersistent chemicals is not straightforward. Because these chemicals have short biologic half-lives, the samples, whether blood or urine, must be collected soon after the exposure in order to appropriately assess the exposure. If the primary exposure medium is the air and the exposure is continuous, a first-morning-void urine sample is probably the best biologic sample for measuring the exposure. However, if the exposure is from a source related to personal grooming (e.g., VOCs from showers or phthalates from personal care products), a first-morning-void urine sample or an early morning blood sample (before showering) would likely miss the exposure from the next day. Rather, a late morning or early afternoon sample would more accurately characterize the daily exposure to these chemicals. Similarly, samples designed to evaluate dietary exposures, such as pesticides, should be collected several hours after mealtimes so that these exposures can be identified.

In general, sample collection for nonpersistent chemical measurements should reflect the residence time of the chemical in each individual matrix. The half-lives of nonpersistent chemicals in blood are typically much less than in urine samples; thus, blood samples may need to be collected within minutes or hours after the exposure, whereas urine samples may be collected several hours or in some instances days after the exposure. Saliva samples will typically mimic blood, whereas meconium samples may provide a longer window for capturing the exposure. Measurements of biomolecular adducts need to consider the lifetime of the biomolecule, rather than the lifetime of the chemical, in the particular matrix; however, more adduct will likely be present immediately after exposure than several weeks after exposure.

### Collecting samples from infants and children.

Difficulty is often encountered when collecting urine samples from infants and children who are not toilet trained. The traditional approach is similar to that in a clinical setting, using an infant urine collection bag. This technique is rather straightforward; however, it is usually bothersome to the child and often requires that the child be given liquids to encourage urination within a given time frame. Encouraging urination with drinks will usually dilute the urine and make the analytical measurement more difficult. Other approaches for urine collection, primarily from cloth diapers or cotton inserts, have also been investigated (Calafat et al. 2004; Hu et al. 2000). Another approach of ongoing investigation is the collection of the target analytes directly from the coagulated gel matrix of disposable diapers (Hu et al. 2004). If proven viable for isolating a broad array of target analytes, this method of collection would be most attractive because it is the least burdensome on the participant and the most logistically practical.

### Temporal variability in urine and blood samples.

The variability of nonpersistent target analyte levels in samples collected from an individual over time is of concern, whether the sample is biologic or environmental. Temporal variability can include the variation of a given chemical in multiple samples collected on a single day or can include variation among days, months, or seasons. For chronic exposures to nonpersistent chemicals, the exposure is repeated; thus, the amount in a given sample would likely represent the average exposure. However, for episodic exposures, the variability is often greater. For urine matrix, a 24-hr urine sample is preferred; however, this can be burdensome on the participant and often logistically difficult. If a 24-hr sample cannot be obtained, a first-morning void is often preferred because the urine is more concentrated and the collection represents a longer window of accumulation (usually > 8 hr). However, a first-morning collection may not be ideal for certain exposures because the timing for capturing the exposure is “off.” For evaluation of daily, monthly, and/or seasonal variations of analyte in urine, sequential samples are often taken days and weeks apart to evaluate how the intraindividual variation over time compares with the interindividual variation and whether an accurate classification of exposure is possible. These studies are important in interpreting the biomonitoring data and should be considered, at some level, in the NCS. These data will help to determine whether multiple samples should be taken and at what intervals. In most instances sampling for nonpersistent chemicals will require multiple samples taken at regular intervals.

## Methodology

### Organic chemicals.

Most methods for measuring organic chemicals in biologic matrices use a sample preparation step to isolate the target chemical(s) from the matrix, an analytical technique with a detection system, data processing, and quality assurance (QA) processes (Needham et al. 2005).

The sample preparation steps are usually the most common source of analytic error, whether systematic or random (Barr et al. 1999). If the chemical is inherently incompatible with the analytic system that follows, a chemical derivatization or reduction procedure may also be required. The addition of steps into the sample preparation procedure usually increases the overall imprecision of the method.

Common analytical techniques for separation of individual chemicals include gas chromatography, high-performance liquid chromatography, or capillary electrophoresis that are coupled in-line to a detection system. Common detection systems include mass spectrometry (MS), electron capture, flame photometric, nitrogen phosphorus, fluorescence, and ultraviolet (UV) absorbance detection. Of the detection systems, mass spectrometers provide the most specificity, and UV absorbance detection usually provides the least (Barr et al. 1999). Most MS-based methods have limits of detection (LODs) in the range of picograms to nanograms per gram of matrix, typically adequate enough to detect levels in the general population when 1–10 g of matrix is used (Table 3). The analytical imprecision usually ranges from 10 to 20%.

Other analytic techniques that are often employed with organic chemicals are immunoassays and bioassays (Biagini et al. 1995; Brady et al. 1989). For these techniques a sample preparation step to isolate the chemical from the matrix may or may not be used. Many are commercially available for selected chemicals. However, their development for a new chemical is a lengthy process that typically requires the generation and isolation of antibodies, then the development of the assay itself. Usually UV, fluorescence, or radioactivity detection is used for the assays. They may be very specific for a given chemical, or they may have a great deal of cross-reactivity that may limit their utility. Their LODs can vary widely; however, many have adequate sensitivity for measuring levels in the general population.

Because organic chemicals are measured using expensive instrumentation and require highly trained analysts, these measurements are usually costly. The most selective and sensitive methods are usually the most complex and can range in cost from $100 to $1500 per sample analyzed (Table 3). However, many of the analyses are multianalyte panels, so the cost per analyte per sample is much more reasonable. Generally, immunoassays are less specific and less complex; therefore, their cost is usually less than $50 per test. However, usually only one chemical can be measured per test, and new chemicals cannot be easily incorporated into the method.

### Inorganic chemicals.

The sample preparation process for inorganic chemicals is typically much simpler than for organic chemicals. In some instances the sample matrix just needs to be diluted with water before analysis. However, special precautions must be taken to avoid contamination, both preanalytically and in the analytic system. For example, prescreened collection materials should be used for sample collection, all analytic supplies should be appropriately free of the target chemicals, and special clean rooms may be required for analysis.

Inorganic chemicals are usually measured using atomic absorption spectrometry (AAS) or inductively coupled plasma (ICP) MS. In some instances a dynamic collision cell may also be used to eliminate potentially interfering salts from the system. When various forms of inorganic chemicals are speciated, such as for arsenic or mercury, the AAS or ICP–MS will be preceded in-line by some chromatographic unit. For lead screening an efficient portable lead analyzer can be used for in-field measurements.

Similar to organic chemicals because expensive instrumentation is used, the analyses are usually costly, ranging from $50 for single chemicals to $250 for multichemical panels (Table 3). The LODs are comparable with those of organic chemicals and are suitable for general population studies. Because the handling of the sample is usually minimal, the precision is usually better, within 5–10%.

### Quality assurance and quality control.

A vital component of all biomonitoring methodology is a sound quality assurance/quality control (QA/QC) program. QA/QC programs typically require strict adherence to protocols and multiple testing procedures that easily allow the detection of systematic failures in the methodology. The requirements for QA/QC are described in detail in Needham et al. (2005).

## Conclusions

As a part of the NCS, many researchers will be competing for the matrices available for biologic measurements. We should refine existing methodology to include as many chemicals as possible using as little blood or urine as possible. In addition we should investigate ways to use more readily available, less invasive matrices. We must consider all matrices and analytes that integrate exposure over longer periods in order to maximize the exposure information gained on an individual using the matrices available during a particular life stage.

Another consideration is the quality and cost of analyses. We should evaluate low-cost techniques such as immunoassays for some applications. In addition to requiring smaller volumes of samples, these analyses are often less expensive and require less training to effectively perform the analyses. Before using these less costly techniques, they should be compared with more commonly used techniques to confirm that quality exposure assessment information—as rated by the method sensitivity, accuracy, specificity, and precision—can be obtained and that the resulting data will be comparable with data existing in the literature.

Generally, persistent organic chemicals are measured more readily in blood-based matrices or other lipid-rich matrices. Maternal measurements serve as good surrogates for fetal exposures and even early childhood exposures if levels are not reduced by breast-feeding. Assessments of exposure to nonpersistent chemicals are the most challenging, but they can be measured in multiple matrix types. Urine is the most commonly used matrix for measurement of these chemicals, but interpretation of the information obtained is often complicated by coexposures, urine dilution, specificity issues, and the temporality of the measurement. To date, no ideal way exists to interpret many of these measurements without the use of additional measures, for example, repeat measurements or environmental measurements. Measurements of metals have been performed in many matrices over the years and are, in general, well understood. Biomonitoring will likely have limited utility in the assessment of exposure to criteria pollutants and bioallergens.

## Figures and Tables

**Table 1:** Importance of various biologic matrices for measuring exposure during the different life stages.

		Fetal period (trimester)			Age (years)		
Matrices	Adult pre-conception	1st	2nd	3rd	0–1	2–3	4–11
**POPs**							
Blood (whole)	1	NA	NA	NA	1	1	1
Blood (serum)	1	NA	NA	NA	1	1	1
Blood (plasma)	1	NA	NA	NA	1	1	1
Urine	3	NA	NA	NA	3	3	3
Saliva	3	NA	NA	NA	NA	3	3
Hair	3	NA	NA	NA	3	3	3
Nails	3	NA	NA	NA	3	3	3
Adipose tissue	1	NA	NA	NA	NA	NA	NA
Feces	3	NA	NA	NA	3	3	3
Semen	3	NA	NA	NA	NA	NA	NA
Breath	3	NA	NA	NA	NA	3	3
Teeth	NA	NA	NA	NA	NA	NA	3
Cord blood	1	1	1	1	3	3	3
Meconium	3	2	2	2	3	3	3
Milk (maternal)	1	1	1	1	1	3	3
Blood (maternal)	1	1	1	1	1	3	3
Urine (maternal)	3	3	3	3	3	3	3
Hair (maternal)	3	3	3	3	3	3	3
Adipose tissue (maternal)	1	1	1	1	1	3	3
**Nonpersistent organic chemicals**							
Blood (whole)	1	NA	NA	NA	1	1	1
Blood (serum)	1	NA	NA	NA	1	1	1
Blood (plasma)	1	NA	NA	NA	1	1	1
Urine	1	NA	NA	NA	1	1	1
Saliva	2	NA	NA	NA	NA	2	2
Hair	3	NA	NA	NA	3	3	3
Nails	3	NA	NA	NA	3	3	3
Adipose tissue	3	NA	NA	NA	NA	NA	NA
Feces	3	NA	NA	NA	3	3	3
Semen	3	NA	NA	NA	NA	NA	NA
Breath	3	NA	NA	NA	NA	3	3
Teeth	3	NA	NA	NA	NA	NA	3
Cord blood	3	3	3	1	3	3	3
Meconium	3	3	2	2	3	3	3
Milk (maternal)	3	3	3	3	2	3	3
Blood (maternal)	3	1	1	1	3	3	3
Urine (maternal)	3	1	1	1	3	3	3
Hair (maternal)	3	3	3	3	3	3	3
Adipose tissue (maternal)	3	3	3	3	3	3	3
**VOCs**							
Blood (whole)	1	NA	NA	NA	1	1	1
Blood (serum)	3	NA	NA	NA	3	3	3
Blood (plasma)	3	NA	NA	NA	3	3	3
Urine	2	NA	NA	NA	2	2	2
Saliva	3	NA	NA	NA	NA	3	3
Hair	3	NA	NA	NA	3	3	3
Nails	3	NA	NA	NA	3	3	3
Adipose tissue	2	NA	NA	NA	NA	NA	NA
Feces	3	NA	NA	NA	3	3	3
Semen	3	NA	NA	NA	NA	NA	NA
Breath	1	NA	NA	NA	NA	1	1
Teeth	3	NA	NA	NA	NA	NA	3
Cord blood	3	3	3	1	3	3	3
Meconium	3	3	3	3	3	3	3
Milk (maternal)	3	3	3	3	2	3	3
Blood (maternal)	3	1	1	1	3	3	3
Urine (maternal)	3	3	3	3	3	3	3
Hair (maternal)	3	3	3	3	3	3	3
Adipose tissue (maternal)	3	3	3	3	3	3	3
**Bioaccumulative inorganic chemicals**							
Blood (whole)	1	NA	NA	NA	1	1	1
Blood (serum)	3	NA	NA	NA	3	3	3
Blood (plasma)	3	NA	NA	NA	3	3	3
Urine	2	NA	NA	NA	2	2	2
Saliva	3	NA	NA	NA	NA	3	3
Hair	2	NA	NA	NA	2	2	2
Nails	2	NA	NA	NA	2	2	2
Adipose tissue	3	NA	NA	NA	NA	NA	NA
Feces	3	NA	NA	NA	3	3	3
Semen	3	NA	NA	NA	NA	NA	NA
Breath	3	NA	NA	NA	NA	3	3
Teeth	3	NA	NA	NA	NA	NA	2
Cord blood	2	2	2	1	3	3	3
Meconium	3	2	2	2	3	3	3
Milk (maternal)	3	3	3	3	3	3	3
Blood (maternal)	1	1	1	1	3	3	3
Urine (maternal)	3	2	2	2	3	3	3
Hair (maternal)	2	2	2	2	3	3	3
Adipose tissue (maternal	3	3	3	3	3	3	3
**Nonbioaccumulative inorganic chemicals**							
Blood (whole)	3	NA	NA	NA	3	3	3
Blood (serum)	3	NA	NA	NA	3	3	3
Blood (plasma)	3	NA	NA	NA	3	3	3
Urine	1	NA	NA	NA	1	1	1
Saliva	3	NA	NA	NA	NA	3	3
Hair	2	NA	NA	NA	2	2	2
Nails	2	NA	NA	NA	2	2	2
Adipose tissue	3	NA	NA	NA	NA	NA	NA
Feces	3	NA	NA	NA	3	3	3
Semen	3	NA	NA	NA	NA	NA	NA
Breath	3	NA	NA	NA	NA	3	3
Teeth	3	NA	NA	NA	NA	NA	3
Cord blood	3	3	3	3	3	3	3
Meconium	3	3	3	3	3	3	3
Milk (maternal)	3	3	3	3	3	3	3
Blood (maternal)	3	3	3	3	3	3	3
Urine (maternal)	3	1	1	1	3	3	3
Hair (maternal)	2	2	2	2	3	3	3
Adipose tissue (maternal)	3	3	3	3	3	3	3
**Criteria pollutants (CO only)**							
Blood (whole)	1	NA	NA	NA	1	1	1
Blood (serum)	3	NA	NA	NA	3	3	3
Blood (plasma)	3	NA	NA	NA	3	3	3
Urine	3	NA	NA	NA	3	3	3
Saliva	3	NA	NA	NA	NA	3	3
Hair	3	NA	NA	NA	3	3	3
Nails	3	NA	NA	NA	3	3	3
Adipose tissue	3	NA	NA	NA	NA	NA	NA
Feces	3	NA	NA	NA	3	3	3
Semen	3	NA	NA	NA	NA	NA	NA
Breath	1	NA	NA	NA	NA	1	1
Teeth	3	NA	NA	NA	NA	NA	3
Cord blood	3	3	3	1	3	3	3
Meconium	3	3	3	3	3	3	3
Milk (maternal)	3	3	3	3	3	3	3
Blood (maternal)	3	1	1	1	3	3	3
Urine (maternal)	3	3	3	3	3	3	3
Hair (maternal)	3	3	3	3	3	3	3
Adipose tissue (maternal)	3	3	3	3	3	3	3
**Bioallergens**							
Blood (whole)	1	NA	NA	NA	1	1	1
Blood (serum)	1	NA	NA	NA	1	1	1
Blood (plasma)	1	NA	NA	NA	1	1	1
Urine	2	NA	NA	NA	2	2	2
Saliva	3	NA	NA	NA	NA	3	3
Hair	3	NA	NA	NA	3	3	3
Nails	3	NA	NA	NA	3	3	3
Adipose tissue	3	NA	NA	NA	NA	NA	NA
Feces	3	NA	NA	NA	3	3	3
Semen	3	NA	NA	NA	NA	NA	NA
Breath	3	NA	NA	NA	NA	3	3
Teeth	3	NA	NA	NA	NA	NA	3
Cord blood	3	1	1	1	3	3	3
Meconium	3	3	3	3	3	3	3
Milk (maternal)	3	3	3	3	3	3	3
Blood (maternal)	3	1	1	1	3	3	3
Urine (maternal)	3	2	2	2	3	3	3
Hair (maternal)	3	3	3	3	3	3	3
Adipose tissue (maternal)	3	3	3	3	3	3	3
**Amount of matrix reasonably obtainable at each life stage**^a^							
Blood (whole)	100	0	0	0	9	22	38
Blood (serum)	40	0	0	0	3.6	8.8	15.2
Blood (plasma)	40	0	0	0	3.6	8.8	15.2
Urine	> 100	0	0	0	1–10	10–20	30–50
Saliva	2	0	0	0	0	1–2	1–2
Hair	0.5–4 g	0	0	0	< 0.5 g	0.5–2 g	0.5–4 g
Nails	*	0	0	0	*	*	*
Adipose tissue	10 g	0	0	0	0	0	0
Feces	10 g	0	0	0	3 g	5 g	1 0g
Semen	2	0	0	0	0	0	0
Breath	*	0	0	0	*	*	*
Teeth	0	0	0	0	0	0	6–10
Cord blood	30–60	30–60	30–60	30–60	NA	NA	NA
Meconium	2 g	2 g	2 g	2 g	NA	NA	NA
Milk (maternal)	> 100	> 100	> 100	> 100	> 100	NA	NA
Blood (maternal)	100	100	100	100	100	100	100
Urine (maternal)	> 100	> 100	> 100	> 100	> 100	> 100	> 100
Hair (maternal)	*	*	*	*	*	*	*
Adipose tissue (maternal) 10 g		10 g	10 g	10 g	0	0	0

Abbreviations: 1, important matrix for most chemicals in category; 2, important matrix for one or two chemicals in category; 3, not an important matrix for assessing exposure for chemicals in the category; NA, matrix not viable for life stage because it cannot be feasibly collected or the chemical cannot typically be measured in the matrix or does not represent exposures in a given life stage;

*, unknown amount. Note that matrices available for assessment in 12- to 21-year-olds are similar to those for adults.

a All units are in milliliters unless otherwise stated.

**Table 2:** Storage requirements and characteristics for biologic matrices and chemical classes.

Chemical class	Chemicals	Storage temperature	Matrix	Matrix stability	Chemical stability	Container	Preservative requirements
POPs	All	–70ºC	Milk	Years	Years	Polypropylene, no glass or Teflon	NA
	All	–70ºC	Serum/plasma	Years	Years	Polypropylene, no glass or Teflon	NA
	All	–70ºC	Adipose tissue	Years	Years	Polypropylene, no glass or Teflon	NA
Nonpersistent organic compounds	All	–70ºC	Urine	Years	Years	Polypropylene or glass	NA
	Phthalates	–70ºC	Serum/plasma	Years	Years	Polypropylene or glass	125 μmol H_3_PO_4_/mL matrix
	Pesticides	–70ºC	Serum/plasma	~ 5 years	Up to 1 year (less for many of the reactive pesticides)	Polypropylene or glass	None
	Others	–70ºC	Serum/plasma	Years	Years	Polypropylene or glass	NA
VOCs		4°C	Whole blood	10 weeks	> 10 weeks	Heat and vacuum-purged glass gray-top Vacutainer; restore sterility	NaF/potassium oxalate
Bioaccumulative metals		4°C	Whole blood	Indefinitely	Indefinitely	Purple-top liquid EDTA Vacutainer; second or third draw	NA
Nonbioaccumulative metals		–20ºC	Urine	Indefinitely	Indefinitely	Prescreened	For Hg, Triton X-100, sulfamic acid
		Room temperature	Hair	Indefinitely	Indefinitely	Zipper bag	NA

**Table 3:** Characteristics of analytical methods for measuring chemical classes in biologic matrices.

Chemical class	Most typical matrices	Methodology used	Detection limits (per gram)	Relative SD (%)	Throughput (samples/day)	Volume for analysis	Cost^a^
POPs	Blood (serum or plasma)	GC-HRMS	fg–pg	15–25	20	2–30 mL	H
	Milk	GC-HRMS	fg–pg	15–25	20	2–30 mL	H
	Adipose tissue	GC-HRMS	fg–pg	15–25	10	1–2 g	H
Nonpersistent organic chemicals	Blood (serum or plasma)	GC-HRMS; HPLC-MS/MS	pg–ng	10–20	30	2–10 mL	H
	Urine	GC-MS/MS; HPLC-MS/MS; immunoassay	pg–ng	10–15	50	1–4 mL	L–M
	Saliva	GC-HRMS; GC-MS/MS; HPLC-MS/MS	pg–ng	10–15	30	1–4 mL	H
	Milk	GC-HRMS; GC-MS/MS; HPLC-MS/MS	pg–ng	10–15	40	1–10 mL	H
VOCs	Blood (whole)	GC-MSD; GC-HRMS	pg	10–20	10–20	5–10 mL	M
	Breath	GC-MSD	ng	10–20	20	10–20 mL	M
Bioaccumulative metals	Blood (whole)	ICP-MS	ng	10–15	40	1–2 mL	L–M
	Hair	ICP-MS	ng	10–15	40		M
Nonbioaccumulative metals	Blood (whole)	ICP-MS	ng	10–15	40	1–2 mL	L–M
	Urine	ICP-MS	ng	10–15	40	1–5 mL	L–M
	Hair	ICP-MS	ng	10–15	40		M

Abbreviations: GC, gas chromatography; HPLC, high-performance liquid chromatography; HRMS, high-resolution mass spectrometry; MS/MS, tandem mass spectrometry; MSD, mass selective detector.

a L, low cost: < $100; M, medium cost: $100–500; H, high cost: > $500.
